# Proteomic Dissection of Endosperm Starch Granule Associated Proteins Reveals a Network Coordinating Starch Biosynthesis and Amino Acid Metabolism and Glycolysis in Rice Endosperms

**DOI:** 10.3389/fpls.2016.00707

**Published:** 2016-05-25

**Authors:** Huatao Yu, Tai Wang

**Affiliations:** ^1^Key Laboratory of Plant Molecular Physiology, Institute of Botany, Chinese Academy of SciencesBeijing, China; ^2^Institute of Chinese Materia Medica, China Academy of Chinese Medical SciencesBeijing, China

**Keywords:** starch granules, starch granules-associated proteins, endosperm, starch biosynthesis, rice

## Abstract

Starch biosynthesis and starch granule packaging in cereal endosperms involve a coordinated action of starch biosynthesis enzymes and coordination with other metabolisms. Because directly binding to starch granules, starch granule-associated proteins (SGAPs) are essential to understand the underlying mechanisms, however the information on SGAPs remains largely unknown. Here, we dissected developmentally changed SGAPs from developing rice endosperms from 10 to 20 days after flowering (DAF). Starch granule packaging was not completed at 10 DAF, and was finished in the central endosperm at 15 DAF and in the whole endosperm at 20 DAF. Proteomic analysis with two-dimensional differential in-gel electrophoresis and mass spectrometry revealed 115 developmentally changed SGAPs, representing 37 unique proteins. 65% of the unique proteins had isoforms. 39% of the identified SGAPs were involved in starch biosynthesis with main functions in polyglucan elongation and granule structure trimming. Almost all proteins involved in starch biosynthesis, amino acid biosynthesis, glycolysis, protein folding, and PPDK pathways increased abundance as the endosperm developed, and were predicted in an interaction network. The network represents an important mechanism to orchestrate carbon partitioning among starch biosynthesis, amino acid biosynthesis and glycolysis for efficient starch and protein storage. These results provide novel insights into mechanisms of starch biosynthesis and its coordination with amino acid metabolisms and glycolysis in cereal endosperms.

## Introduction

Cereal seeds consist of embryo, endosperm, and pericarp, with endosperm as the main part where reserve materials accumulate during development. Starch is the main reserve material, and represents 85% dry weight of cereal endosperms, thus being main food source for humans and livestock worldwide (Ruuska et al., 2002). Starch is composed of amylose and amylopectin glucan polymers which are biosynthesized and together packaged to form the semicrystalline starch granules in amyloplasts of cereal endosperms (Sabelli and Larkins, 2009; Zeeman et al., 2010). During cereal endosperm development process, starch is synthesized and accumulated by the coordinated action of starch biosynthetic enzymes, including ADP-glucose pyrophosphorylase (AGPase), starch synthase, starch branching enzyme (SBE), starch debranching enzyme (DBE), and plastidial starch phosphorylase (Pho1; Jeon et al., 2010; Zeeman et al., 2010). Starch synthase can be further divided into granule bound starch synthase (GBSS) and soluble starch synthase (SS). GBSS is involved in amylose synthesis. SS is required for amylopectin synthesis in the combination with SBEs and DBEs. DBE can be further divided into isoamylase (ISA) and pullulanase (PUL). UDP-glucose pyrophosphorylase (UGPase) has also been reported to be related with starch metabolism in cereal endosperms (Mechin et al., 2007; Yu et al., 2012).

Most of these enzymes are distributed between soluble form in amyloplast stroma and insoluble starch granule associated form (Regina et al., 2005; Grimaud et al., 2008; Zeeman et al., 2010). Studies have analyzed proteome of soluble fractions of rice, maize, barley and wheat seeds or endosperms at different developmental stages (Finnie et al., 2002; Vensel et al., 2005; Mechin et al., 2007; Xu et al., 2008, 2010). Most of identified starch biosynthesis enzymes in maize and rice showed increased expression during development, in accordance with the starch accumulation profiles. These studies also showed the coordination of different metabolisms is essential for efficient starch biosynthesis and storage (Mechin et al., 2007; Xu et al., 2008, 2010). Because direct association with starch granules, starch granule-associated proteins (SGAPs) are important for further understanding the mechanisms underlying starch biosynthesis and its coordination with other metabolism processes. Several studies have analyzed SGAPs of barley, maize and wheat (Boren et al., 2004; Grimaud et al., 2008; Cao et al., 2015). The analysis of wheat SGAPs revealed 13 unique proteins, and demonstrated activity of granule-associated starch biosynthesis enzymes was correlated with size of starch granules (Cao et al., 2015). The study of maize endosperms revealed the starch granule association of SSIII, BEI, BEIIa, BEIIb, GBSS, and SSI, and phosphorylation modification of GBSS, SBEIIb, and Pho1 occurred in the granule (Grimaud et al., 2008). Among these enzymes, the absence of a protein could affect starch granule-association of another protein (Grimaud et al., 2008). This suggests their coordination in function. Therefore, study on dynamic changes of SGAPs during endosperm development will provide novel insights into the mechanisms underlying starch biosynthesis and its coordination with other metabolic processes, but the study is lacking.

Rice is an important monocot and cereal model plant and one of the most important crops. We have characterized cellular features and proteomes of developing seeds and endosperms of rice and revealed the coordination of different metabolic and cellular processes is essential to immobilize nutrients for starch biosynthesis (Xu et al., 2008, 2010). Here, we proteomically analyzed SGAPs of developing rice endosperms at 10, 15, and 20 days after flowering (DAF) using prepared starch granules. We revealed 115 developmentally changed SGAPs. These proteins were implicated in 9 functional categories and overrepresented by starch biosynthesis enzymes which mainly function in polyglucan elongation and granule structure trimming. Most proteins increased abundance as the endosperm developed. Proteins implicated in starch biosynthesis, amino acid biosynthesis, glycolysis, protein folding, and pyruvate orthophosphate dikinase (PPDK) pathways were predicted in an interaction network. The network represents an important mechanism to orchestrate carbon partitioning among these processes for efficient starch and protein storage.

## Materials and methods

### Plant materials and sampling

The rice cultivar Zhonghua 10 (*Oryza sativa* L. ssp. *japonica*) was grown in Beijing. At flowering stage, rice plants were transferred into a phytotron growth chamber with 12-h/30°C light and 12-h/24°C dark. Superior caryopses (seeds) were labeled at noon of flowering day as described (Xu et al., 2008; Yu et al., 2012). These labeled caryopses were harvested and dehusked at 10, 15, and 20 DAF, respectively, and used immediately or stored at −80°C until use after frozen in liquid nitrogen.

### Starch granule preparation

Transections of endosperms at 10, 15, and 20 DAF were observed under a stereomicroscope (Leica S8APO) equipped with a digital camera (ProgResC5cool, Jenoptik) to characterize the developmental status of endosperms.

Endosperms were ground in ice-cold extraction buffer (50 mM Tris-HCl, pH 8.0, 1 mM EDTA, 2 mM PMSF), followed by filtration through 250-mesh cell strainer. The filtrate was further fractionated on Percoll (100%) by use of centrifugation at 500 × *g* for 30 min at 4°C. The resultant pellet was washed with washing buffer (50 mM Tris-HCl, pH 8.0, 1 mM EDTA, 2 mM PMSF, 10% (v/v) Glycerol, 0.2% (v/v) Triton X-100) at 4°C for 7 times with 10 min for each, followed by washing by water and cold acetone in sequence for 3 and 2 times each, and air-dried. Starch granules were then treated with 0.01 M NaOH at a ratio of 25 mg/mL for 15 min with continuous vibrating, collected by use of centrifugation at 4000 × *g* for 5 min at 25°C, washed with water, and used for protein extraction. Four independent starch granule preparations were performed for each developmental stage.

### SGAPs extraction and one-dimensional electrophoresis

SGAPs were extracted as described with modifications (Bancel et al., 2010). In brief, 0.3 g starch granules were mixed with 10 mL SDS buffer (62.5 mM Tris-HCl, pH 8.7, 2% (w/v) SDS, 10 mM DTT), incubated at 100°C for 10 min under continuous agitation, cooled on ice for 10 min, then centrifuged at 10,000 × *g* for 15 min at 25°C. The extraction process was repeated again. Combined supernatant was added one volume 20% TCA (w/v) in acetone, incubated at −20°C overnight followed by centrifugation at 37,000 × *g* for 20 min at 4°C. The protein pellet was washed twice with ice-cold 80% acetone and air-dried. Resultant protein pellet was dissolved in dense SDS buffer (0.1 M Tris-HCl, pH 8.0, 30% (w/v) sucrose, 2% (w/v) SDS, 10 mM DTT), mixed with equal volume of Tris-saturated phenol (pH 8.0, Sigma), vortexed for 10 min and centrifuged at 20,000 × *g* for 10 min at 20°C. The upper phenol phase was added 5 volumes of ice-cold 0.1 M ammonium acetate in methanol, incubated at −20°C overnight and centrifuged at 37,000 × *g* for 20 min at 4°C to collect proteins. Proteins were washed with cold 0.1 M ammonium acetate in methanol, subsequently with cold 80% acetone, air-dried, and finally dissolved in lysis buffer (7 M urea, 2 M thiourea, 4% (w/v) CHAPS, 20 mM Tris-HCl, pH8.5) at room temperature.

Four independent starch granule preparations were used for 4 independent SGAPs extraction. The proteins were quantified according to the Bradford method by DU640 UV-visible spectrophotometry (Beckman) with bovine serum albumin as a standard. For examination of SGAP patterns, 10 μg SGAPs were loaded into each lane, and resolved with sodium dodecyl sulfate-polyacrylamide gel electrophoresis (SDS-PAGE) in 12.5% gel.

### Two-dimensional differential in-gel electrophoresis (2D-DIGE), image analysis, and protein identification

2D-DIGE, image analysis, and protein identification were all performed as described (Yu et al., 2012). Briefly, SGAP samples of the 3 developmental stages with 4 biological repeats each and the internal standard prepared by mixing equal amounts of all analyzed samples were labeled with Cydye (GE Healthcare) (Figure S1A, Supporting Information). These labeled protein samples were assigned to 6 DIGE gels (Figure S1A, Supporting Information), and separated with 2-D electrophoresis involving the utilization of 24-cm, pH 3-10 NL IPG strip (GE Healthcare). Fluorescent images of gels were acquired with Typhoon 9410 scanner (GE Healthcare) and analyzed with DeCyder 7.0 software (GE Healthcare). Spots reproducible in the 18 images (derived from the 6 DIGE gels, each gel contained two different samples and one internal standard sample) (Figure S1B, Supporting Information) were used for further analysis. Differential expression of protein spots were determined by analysis of variance (ANOVA) (*p* < 0.05) and Student's *t*-test (*p* < 0.05), and false discovery rate (FDR) correction was used. Differentially expressed spots were picked from Coomassie Brilliant Blue (CBB) stained gels which contained 1 mg internal standard proteins. Picked spots were in-gel digested with trypsin (Roche), and then proteins were identified with UltrafleXtreme matrix-assisted laser desorption ionization time-of-flight/time-of-flight mass spectrometry (MALDI-TOF/TOF-MS) (Bruker Daltonics, Germany). Combined MS and MS/MS results were submitted to Mascot engine 2.4.1 (http://www.matrixscience.com) in BioTools 3.2 software (Bruker Daltonics), and searched against the NCBInr protein database (http://www.ncbi.nlm.nih.gov/; NCBInr 20130727; 31244244 sequences; 10788889170 residues) with the taxonomy selection of *Oryza sativa* (135576 sequences). Parameters for search were set as follows: enzyme, trypsin; max missed cleavages, 1; fixed modifications, Carbamidomethyl (C); variable modifications, Oxidation (M); peptide mass tolerance, 100 ppm; fragment mass tolerance, 0.5 Da; mass values, Monoisotopic; charge state, 1^+^. Proteins with significant hits (at least one unique peptide of *p* < 0.05 matched) were recorded.

### Bioinformatics analysis

Protein annotation and classification were mainly according to the NCBI protein database (http://www.ncbi.nlm.nih.gov/) and Uniprot database (http://www.uniprot.org/). Unique protein (non-redundant protein) identification was performed as previously described (Yu et al., 2012). K-means cluster analysis was performed with the MeV 4.9 software (http://www.tm4.org/index.html). Protein interaction network was built with the STRING 10 tool (http://string-db.org/).

## Results

### Characteristics of rice endosperms

**Figure 1:**
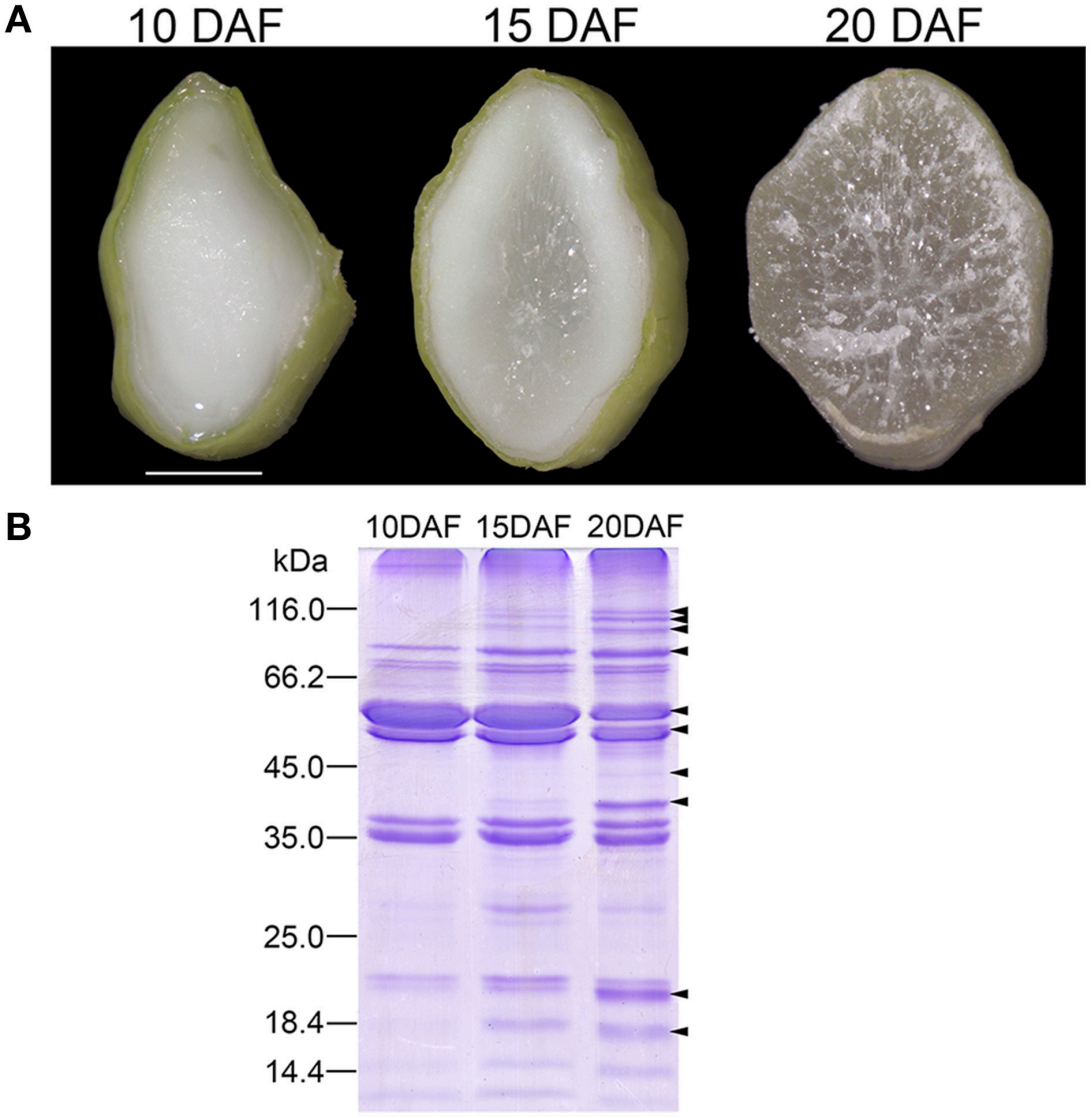
**Cellular features of rice endosperms at 10, 15, and 20 days after flowering (DAF) and sodium dodecyl sulfate-polyacrylamide gel electrophoresis (SDS-PAGE) patterns of starch granule associated proteins (SGAPs) of the endosperms**. **(A)** Transverse images of endosperms at 10, 15, and 20 DAF. Scale bar = 1.5 mm. **(B)** SDS-PAGE patterns of SGAPs from starch granules prepared from endosperms at the three stages. Arrows showing protein bands obviously changed during endosperm development.

Rice seeds on upper primary branches or lower secondary branches of the rachis are defined as superior or inferior seeds, respectively, and superior seeds were selected in this study because of their synchronous development (Ishimaru et al., 2003). Rice seed mainly accumulated starch between 10 and 20 DAF (Xu et al., 2008). We observed transections of 10, 15, and 20 DAF endosperms to reveal the developmental characteristics. Endosperm transection of 10 DAF was uniformly milk-white (Figure 1A). Central region of the section became translucent at 15 DAF, indicating the starch-fill and desiccating endosperm (Figure 1A). Starch deposition and granule packaging completed in this region (Xu et al., 2010). The translucent region spread to the whole section at 20 DAF (Figure 1A). Therefore, endosperms at the three stages represented different physiological status of starch biosynthesis and packaging: on-going, half-finished, and complete-finished.

### SGAPs expression profiles and identification of differentially expressed proteins

**Figure 2:**
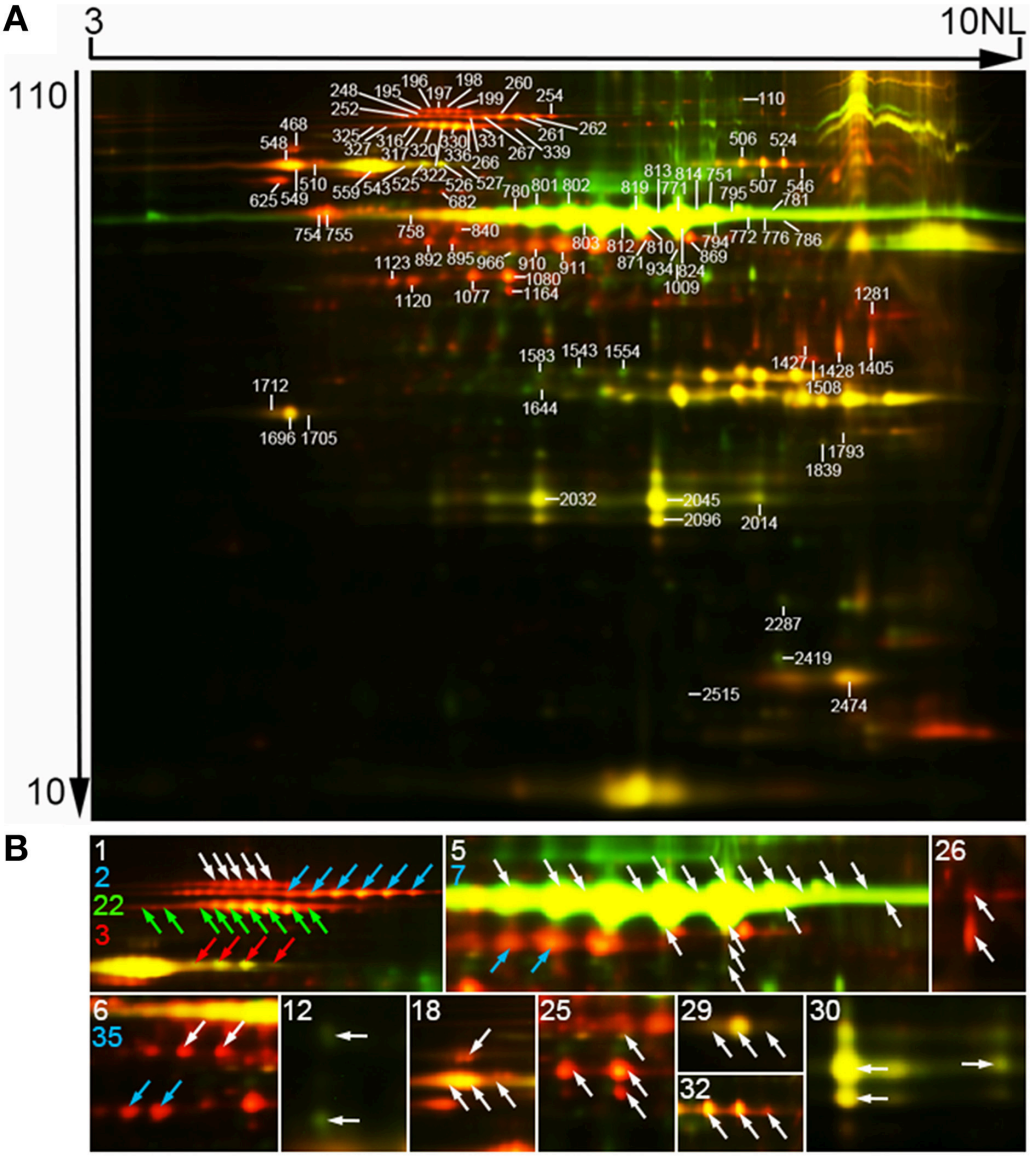
**Representative two-dimensional differential in-gel electrophoresis (2D-DIGE) images of SGAPs**. **(A)** Representative image. Molecular mass (in kilodaltons) and pI of proteins were shown at the respective left and top of the image. Numbered spots representing identified differentially expressed protein spots. Original data of these spots were in Tables S1, S2, S6. **(B)** Close-up of isoforms of proteins from one protein-containing spots. Characters representing unipro number. Arrows colored same as a character indicating isoforms of the proteins. 1, plastidial starch phosphorylase (Pho1); 2, pullulanase (PUL); 3, soluble starch synthase I (SSI); 5, granule bound starch synthase (GBSS); 6, alanine aminotransferase; 7, ADP-glucose pyrophosphorylase 51 kD subunit (AGPase S2a); 12, glutelin; 18, heat shock 70 protein; 22, pyruvate orthophosphate dikinase 1 (PPDKB); 25, phosphoglycerate kinase; 26, glyceraldehyde-3-phosphate dehydrogenase; 29, desiccation-related protein PCC13-62; 30, germin-like protein 3; 32, chloroplastic outer envelope membrane protein; 35, actin. Raw data of isoforms and corresponding unipros were in Tables S4, S5, Supporting Information.

We firstly evaluated difference in expression patterns of SGAPs from 10, 15, and 20 DAF endosperms using SDS-PAGE. The analysis revealed several SGAP bands changed obviously in developing endosperms from 10 to 20 DAF, most of which showed increased abundance as endosperm developed (Figure 1B). To solve the expression patterns of SGAPs and reveal differentially expressed proteins in 10, 15 and 20 DAF endosperms, we used 2D-DIGE with pH 3-10 NL strips to separate SGAPs (Figure 2A, Figure S1B, Supporting Information). This experiment generated 18 images from 6 DIGE gels with 4 independent biological repeats for each distinct sample (Figure S1, Supporting Information). The quantitation and statistics analysis of protein spots across these images were performed, and differentially expressed protein (DEP) spots were determined by ANOVA and Student's *t*-test (*p* < 0.05).

**Figure 3:**
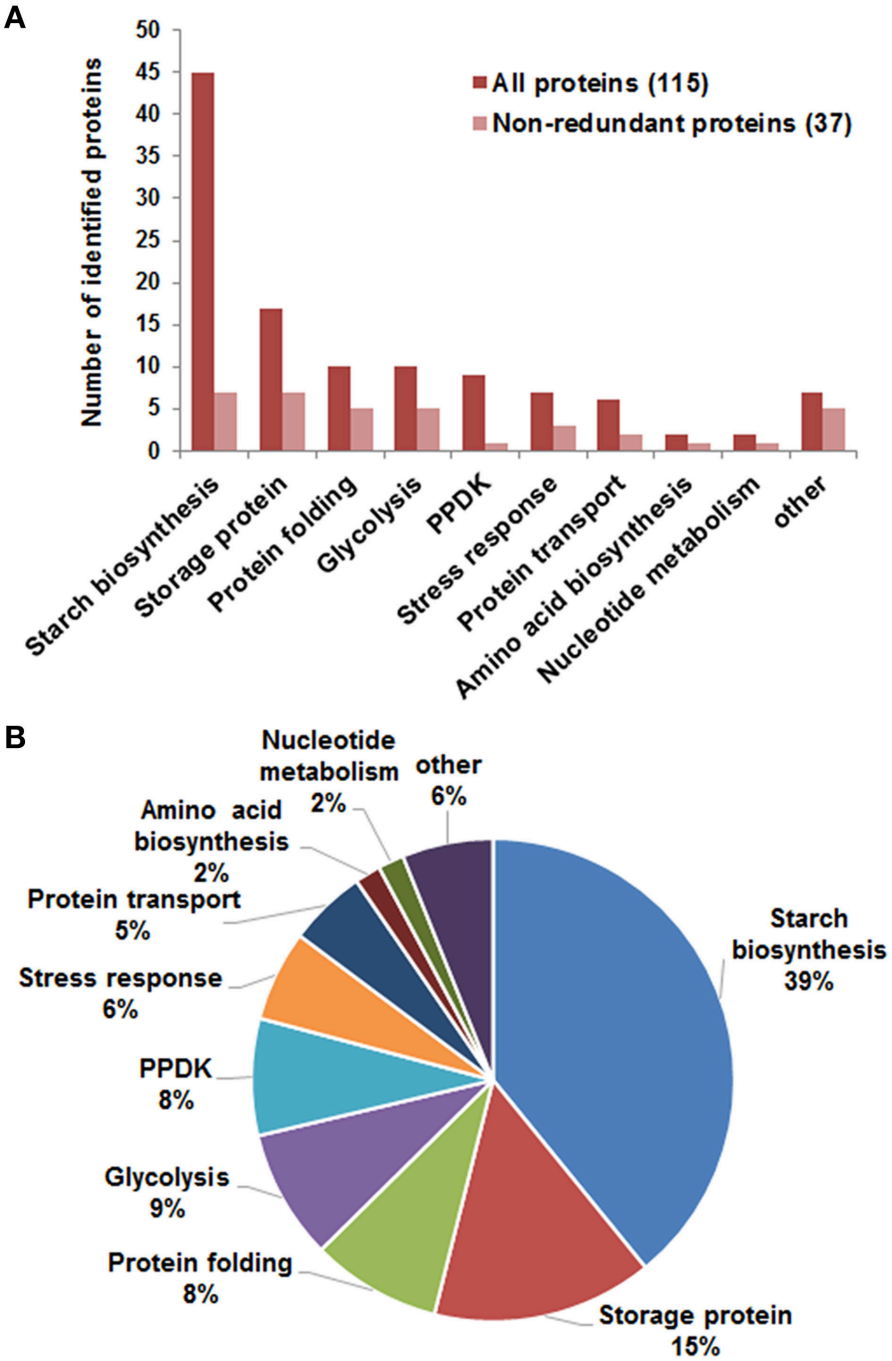
**Function categories of identified differentially expressed proteins**. **(A)** Function classification of identified 115 proteins (red) representing 37 unipros (light red). Information involving protein identification was in Table S2, and function features in Table S6, Supporting Information. **(B)** Percentage of proteins in each category to the total 115 proteins.

Mass spectrometry analysis identified 97 DEP spots (Figure 2A, Figure S2, Tables S2, S3, Supporting Information). Among them, 80 spots contained only 1 protein each, 16 contained 2 proteins each, and 1 contained 3 proteins. Therefore, we totally identified 115 proteins, representing 37 unique proteins (unipros) (Table S2, Supporting Information), thus indicating high number of isoforms for most unipros. According to database annotation, the 115 proteins were classified into 9 function categories, which were implicated in biosynthesis of starch and amino acids, nucleotide metabolism, protein folding and transport, glycolysis and stress response, and one “other” category which contained proteins not clearly grouped (Figure 3A). The largest group was starch biosynthesis, which contained 39% of these identities (Figure 3B, Table S2, Supporting Information), being consistent with the active starch synthesis and granule packaging process in the endosperm.

### Isoforms of proteins

Isoforms of proteins are usually identified in two-dimensional (2D) gel based proteomic research as a result of posttranscriptional gene transcripts splicing or posttranslational protein modification (Venu et al., 2011; Cao et al., 2015). In this study, 24 (65%) of the 37 identified unipros had isoforms from 2 (8 unipros) to 22 (GBSSI) (Table S4, Supporting Information). We further investigated isoforms expression profiles with excluding those from spots having 2 or more identities. This analysis involved 69 identities corresponding to 15 unipros (Table S5, Supporting Information). Among the 15 unipros, nine (unipro 1, 2, 3, 6, 7, 22, 29, 32, and 35) had isoforms with same molecular mass but different pI values, thus these isoforms appeared horizontal distribution on 2D-DIGE image (Figure 2B). Two (unipro 12 and 26) had isoforms with similar pI values but different molecular mass (Figure 2B). Four (unipro 5, 18, 25, and 30) had isoforms with both the above conditions (Figure 2B). Data searching found that 5 unipro-encoding genes have more than one alternative splicing transcripts in rice genome annotation project database (http://rice.plantbiology.msu.edu/index.shtml) and 4 unipros have posttranslational modifications in Uniprot database (http://www.uniprot.org/) (Table S5, Supporting Information). Studies had revealed phosphorylation of starch granule-associated GBSSI and SSI from wheat endosperm, and phosphorylation made isoforms of the two proteins appeared same molecular mass with different pI values (Bancel et al., 2010; Cao et al., 2015). Together, these results suggest phosphorylation modification along with alternative splicing may be important mechanisms to generate isoforms of SGAPs at least in rice endosperms.

All isoforms of 12 of the analyzed 15 unipros had significantly positively correlated expression profiles (*p* < 0.05) (Table S5, Supporting Information). While 3 of the 6 isoform pairs of unipro 25 (Phosphoglycerate kinase) were significantly correlated (Table S5, Supporting Information). Only GBSSI (unipro 5) had both positively and negatively correlated isoform pairs (Table S5, Supporting Information).

### Coexpression patterns of differentially expressed proteins

**Figure 4:**
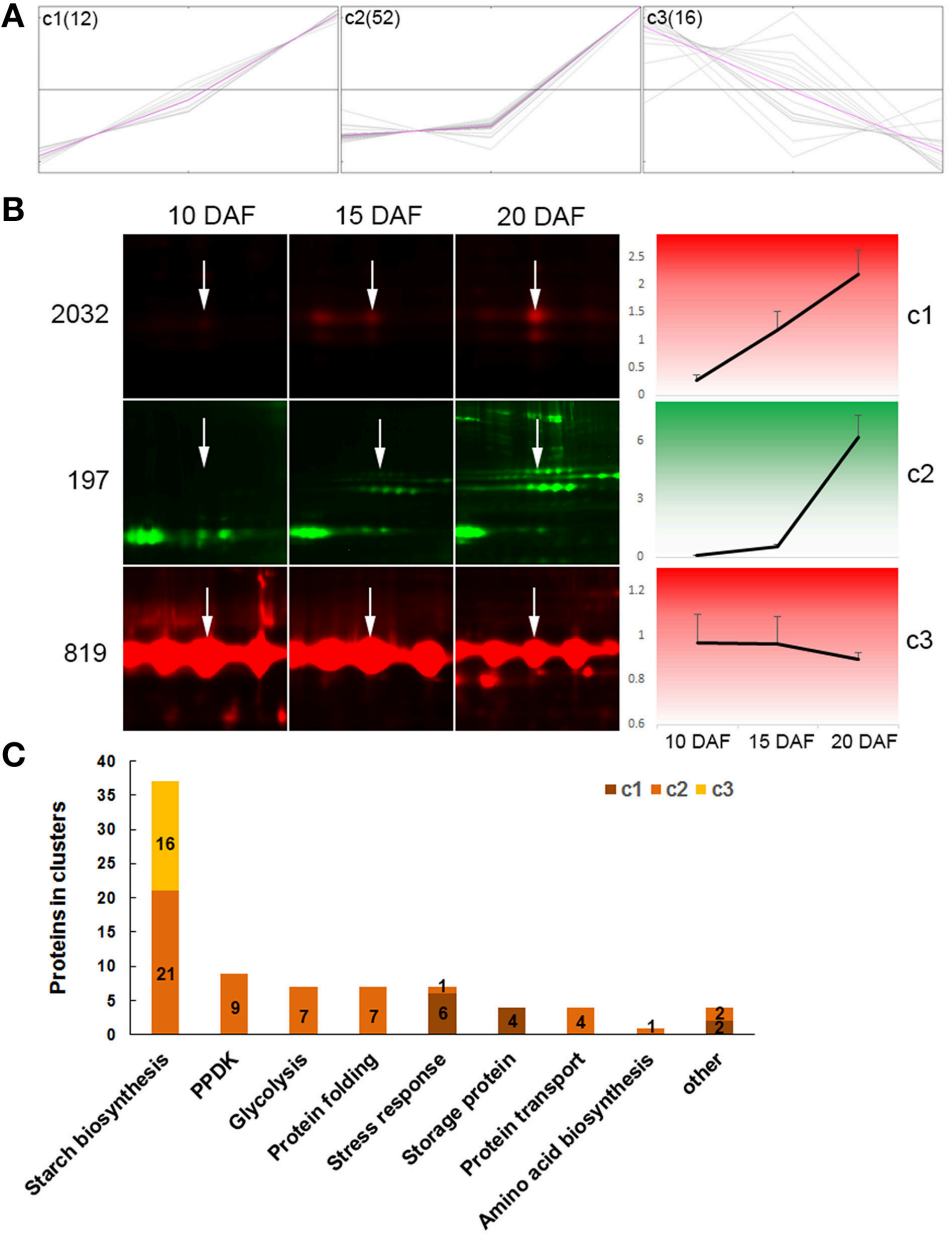
**Cluster analysis of differentially expressed proteins**. Only proteins from one-protein containing spots were included. **(A)** Proteins were organized into 3 clusters with the MeV 4.9 software. Expression abundance of proteins were first normalized, then used in cluster analysis. Gray lines representing expression profiles of individual proteins in a cluster, and pink lines representing concentriods of each cluster. **(B)** Expression profiles of three protein spots as examples: spot 2032 for c1, spot 197 for c2, and spot 819 for c3. White arrows show the indicated spot in the distinct developmental stage. **(C)** Distribution of proteins from different function categories in the three clusters.

We used the k-means cluster algorithm to analyze coexpression patterns of 80 DEPs from spots which had only one protein each, with exclusion of 17 spots having 2 or more proteins (Table S6, Supporting Information). The analysis revealed 3 distinct expression patterns (c1, c2, and c3) (Figure 4A, Table S6, Supporting Information). Proteins in c1 (12 proteins) were linearly increased in abundance from 10 to 20 DAF; proteins in c2 (52 proteins) showed little increase from 10 to 15 DAF, and then drastic increase in abundance from 15 to 20 DAF (Figure 4A). While proteins in c3 (16 proteins) appeared linearly decreased abundance from 10 to 20 DAF, being opposite to the expression pattern of proteins in c1 (Figure 4A). These expression patterns were clearly observable in 2D-DIGE images and exemplified with spot 2032 for c1, spot 197 for c2, and spot 819 for c3 (Figure 4B).

Furthermore, we observed expression patterns of proteins in given function categories (Figure 4C, Table S6, Supporting Information). Among the 37 starch biosynthesis associated proteins, 21, which were mainly isoforms of Pho1, PUL, SSI, UGPase and AGPase, were in c2; while the remaining 16 were in c3 and represented all proteins of this cluster (Figures 4A,C). All the 16 proteins in c3 were isoforms of GBSSI (Table S6, Supporting Information). PPDK, and proteins involved in glycolysis, protein folding and transport, and amino acid biosynthesis were all in c2. All the 4 storage proteins and most (6/7) of stress response-related proteins were in c1. These data demonstrated proteins in a given function group coexpressed.

### Protein interaction network

**Figure 5:**
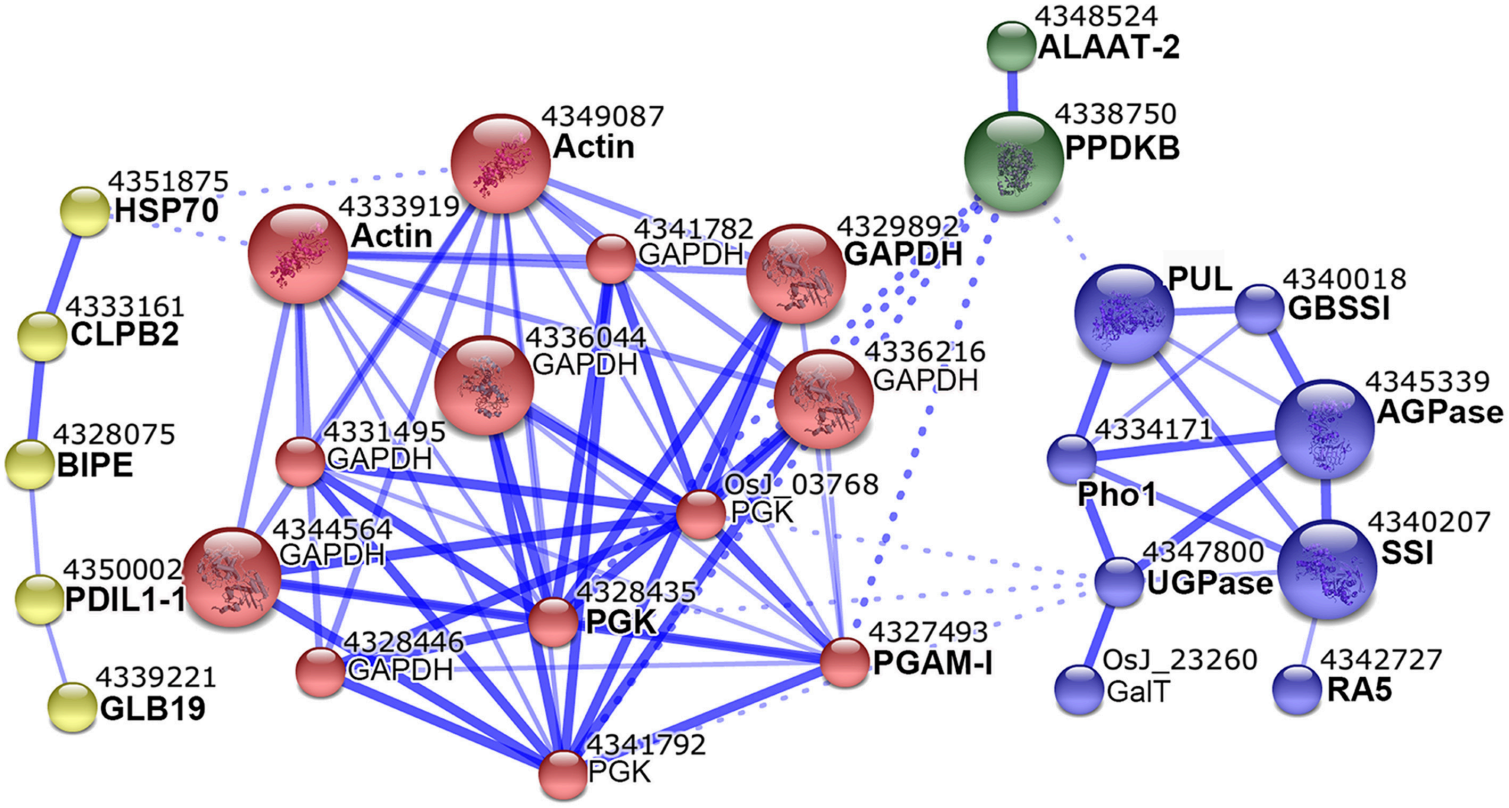
**Interaction network of differentially expressed proteins**. Proteins identified from one protein-containing spots were analyzed with STRING 10 tool. Interactions at medium confidence (score > 0.4) and evidences from experiments, database, and text mining were considered. Nodes with no, or scattered interactions were excluded. The network were grouped into 4 modules in different colors with the MCL clustering algorithm. Solid and dashed lines represented inner and inter-group interactions, respectively. Each node corresponded to one protein, and protein names were labeled under the node numbers. Proteins with names in bold were our input, and those in regular were predicted functional partners.

To further understand the biological function and possible relation of these DEPs, we analyzed their interaction network with the STRING 10 tool. The 80 DEPs used in this analysis corresponded to 31 non-redundant GI accessions (Table S6, Supporting Information), and amino acid sequences of the 31 GI accessions were submitted to STRING 10 tool to retrieve protein interaction network. With a medium confidence level (score > 0.4) and evidences from experiments, database, and text mining considered, we revealed that 19 of the 31 GI accessions were within one network which also contained 9 predicted functional partners (Figure 5).

The network could have 4 modules (Figure 5). The first module (yellow) contained 5 proteins, and 4/5 including heat shock 70 protein (HSP70), chaperone protein ClpB2 (CLBP2), endosperm lumenal binding protein (BIPE), and protein disulfide isomerase-like 1-1 (PDIL1-1) were implicated in protein folding (Table S6, Supporting Information). The second module (red) contained 13 proteins, of which glyceraldehyde-3-phosphate dehydrogenase (GAPDH), 3 phosphoglycerate kinase (PGK), and 2,3-bisphosphoglycerate-independent phosphoglycerate mutase (PGAM-I) were all in glycolysis category (Table S6, Supporting Information). The third module (green) contained PPDKB and alanine aminotransferase 2 (ALAAT-2). The fourth module (blue) contained 8 proteins, of which PUL, Pho1, SSI, GBSSI, AGPase and UGPase were all involved in starch biosynthesis (Table S6, Supporting Information). This network suggested probable coordination between starch biosynthesis, glycolysis, PPDK, and amino acid biosynthesis by the assistance of protein folding proteins.

## Discussion

### Starch granule-associated starch biosynthesis enzymes are mainly involved in polyglucan chains elongation and granule structure trimming

Grain filling of rice seeds was mainly during 6–20 DAF (Xu et al., 2008). The seed kept enlarging and reached a size close to the mature seed at 10 DAF. The seed weight increased during development and didn't increase any more after 20 DAF. Therefore, rice seeds mainly accumulated storage materials during 10–20 DAF. We dissected SGAP proteome of developing rice endosperms from 10 to 20 DAF, and revealed 80 differentially expressed SGAPs in the developing endosperm. 37 of them (46.25%) were starch biosynthesis-associated, such as GBSSI, SSI, AGPase, UGPase, Pho1, and PUL with each having multiple isoforms (Table S6, Supporting Information). GBSSI is responsible for extending polyglucan chains of amylose (Jeon et al., 2010; Zeeman et al., 2010), and SSI accounted for about 70% of the total SS activity in rice endosperms for elongating short glucan chains of amylopectin (Jeon et al., 2010; Zeeman et al., 2010). Therefore, GBSSI and SSI play important roles in polyglucan chains elongation. Consistent with our results, studies of other cereal endosperms also revealed the two proteins are granule internals (Boren et al., 2004; Grimaud et al., 2008; Bancel et al., 2010; Wang et al., 2013). This indicated their tight association with the granule. AGPase and UGPase are directly or indirectly responsible for supplying donors for polyglucan chain elongation which is mainly catalyzed by GBSS and SSI in rice endosperms (Koch, 2004; Jeon et al., 2010; Zeeman et al., 2010). AGPase has been reported to be copurified with SSIII (Hennen-Bierwagen et al., 2009). As a result, granule association of AGPase is probably due to its interaction with starch synthases which are tightly granule-associated (see above). Pho1 functions at granule surface to catalyze the reversible transfer of glucosyl from the polyglucan chains (Jeon et al., 2010; Zeeman et al., 2010). PUL is a DBE functioning in amylopectin synthesis (Jeon et al., 2010; Zeeman et al., 2010). Therefore, both Pho1 and PUL can modify synthesized polyglucan to trim and modify granule structure and make the granule package correctly. Together, these results show starch granule-associated starch biosynthesis enzymes are mainly involved in polyglucan elongation and starch granule trimming during endosperm development.

To evaluate the presence of starch synthesis enzymes in starch granule-associated and/or soluble forms, we retrieved the data of starch synthesis-related proteins which were identified in soluble proteomes of developing rice seeds (Xu et al., 2008) and endosperms (Xu et al., 2010) (Table S7, Supporting Information). Results showed that GBSSI and SSI were only detected in starch granule-associated form; AGPase, UGPase, PUL and Pho1 in the two forms; Isoamylase I (ISA I), isoamylase III (ISA III) and plastidic phosphoglucomutase (PPGM) only in soluble form (Table 1). ISA is another type of DBEs, and has a role in determining granule number in the starch granule initiation step of barley, potato and rice (Burton et al., 2002; Bustos et al., 2004; Kawagoe et al., 2005). So ISA is more probably associated with starch granule initiation cores. PPGM functions in maintaining a Glc-6-P/Glc-1-P pool for starch synthesis when source supply is deficient in rice seed development (Xu et al., 2008). SBE was not detected in previous soluble proteome of rice endosperms and this starch granule-associated proteome studies, this may be due to that it was not easily resolved in 2-D gel or not differentially expressed during endosperm development. These data suggest that soluble starch biosynthesis enzymes may be mainly involved in starch granule initiation, trimming and maintenance of a Glc-6-P/Glc-1-P pool, while starch granule-associated enzymes mainly in polyglucan elongation and starch granule trimming in developing endosperm.

**Table 1:** **Comparison of differentially expressed starch granule-associated and soluble starch metabolism enzymes in developing rice endosperms**.

**Identification**	**Proteins**
Identified in both forms	ADP-glucose pyrophosphorylase (AGPase)
	UDP-glucose pyrophosphorylase (UGPase)
	alpha 1,4-glucan phosphorylase L isozyme (Pho1)
	Pullulanase (PUL)
Soluble	Isoamylase I (ISA I)
	Isoamylase III (ISA III)
	Plastidic phosphoglucomutase (PPGM)
SGA	Granule-bound starch synthase (GBSSI)
	Starch synthase I (SSI)
Not identified in both forms	Starch branching enzyme (SBE)

*The soluble differentially expressed starch metabolism enzymes were obtained from the published data of developing rice seed and endosperm proteomes (Xu et al., 2008, 2010) (Table S7, Supporting Information)*.

All the 6 identified starch granule-associated starch biosynthesis enzymes except GBSSI increased abundance in developing endosperms (c2 in Figure 4, Table S6, Supporting Information). These proteins have multiple isoforms, distributing horizontally in 2-D images (Figure 2B). The features are compatible with phosphorylation modification (Bancel et al., 2010; Cao et al., 2015). Furthermore, the isoforms of these proteins mainly displayed positively correlated expression (Table S5, Supporting Information). This suggests phosphorylation modification may be important mechanism to diversify functions of these enzymes (Tetlow et al., 2004, 2008; Hennen-Bierwagen et al., 2009; Liu et al., 2009). Correspondingly, soluble starch synthesis enzymes also displayed increase in abundance during the development (Table S7, Supporting Information) (Xu et al., 2008). This is consistent with the storage material accumulation in rice seeds and changes in starch granule packaging station at this developmental stage (Xu et al., 2008; Figure 1A). Therefore, starch synthesis and accumulation in developing rice endosperm may be achieved by up-regulation of starch biosynthesis enzymes and increased granule association of starch biosynthesis enzymes implicated in polyglucan elongation and granule trimming.

### Coordinated carbon partitioning among starch biosynthesis, amino acid biosynthesis and glycolysis around starch granules

The interaction network of differentially expressed SGAPs consists of four modules which mainly involved in starch biosynthesis, glycolysis, PPDK and amino acid biosynthesis, and protein folding (Figure 5). In the starch biosynthesis module, AGPase, which catalyzes reversible conversion from glucose-1-phosphate (Glc-1-P) and ATP to ADP-Glucose (ADP-Glc) and inorganic pyrophosphate (PPi), is present in both cytosol and amyloplast in cereal endosperms with the most activity at cytosol and a small portion at amyloplast (Jeon et al., 2010). The cytosol ADP-Glc is transported into amyloplast for starch biosynthesis together with the amyloplast-produced ADP-Glc (Jeon et al., 2010). AGPase activity was reported at dynamic equilibrium in barley amyloplast (Tiessen et al., 2012). This equilibrium not only permits production of ADP-Glc and PPi, but also permits the reverse reaction in the amyloplast. For the reverse reaction, substrate ADP-Glc is mainly imported from cytosol (Jeon et al., 2010), and PPi can be produced by amyloplastidial PPDK. Consistent with the involvement of AGPase and PPDK in one network (Figure 5), PPDK is reported copurified with AGPase in maize amyloplast extracts (Hennen-Bierwagen et al., 2009). PPKD catalyzes the reversible conversion of pyruvate, inorganic phosphate (Pi) and ATP into phosphoenolpyruvate (PEP), AMP and PPi, thus can supply PPi for AGPase-catalyzed reaction in amyloplast. Besides ADP-Glc, Glucose-6-phosphate (Glc-6-P) is also imported into cereal amyloplast, then conversed to Glc-1-P (Jeon et al., 2010). Pho1 also produces Glc-1-P by reversibly transferring glucosyl from the polyglucan chains (Jeon et al., 2010; Zeeman et al., 2010). AGPase-mediated reversible conversion of Glc-1-P and ADP-Glc keeps balance between the two compounds by coordination with PPDK. PPDK may be implicated in amino acid biosynthesis by PEP and pyruvate (Mechin et al., 2007). Glycolysis converts Glc-1-P to series of metabolites, most of which can be used for amino acid biosynthesis. Correspondingly, almost all enzymes (proteins) involved in these metabolisms displayed up-regulated expression during endosperm development (Figures 4A,C, c2), and this is consistent with the storage material accumulation in rice seeds and changes in starch granule packaging station at this developmental stage (Xu et al., 2008; Figure 1A). Together, these data suggested that protein interaction network represents an important mechanism to orchestrate carbon partitioning among starch biosynthesis, amino acid biosynthesis and glycolysis for efficient starch and protein storage by the assistance of protein folding proteins.

## Conclusions

We dissected SGAPs of developing endosperms from 10 to 20 DAF, the fast accumulation stage of storage materials, and revealed a set of SGAPs, of which 65% have isoforms. The SGAPs dataset displayed important skew onto starch biosynthesis enzymes which were mainly involved in polyglucan elongation and granule structure trimming. Almost all proteins involved in starch biosynthesis, amino acid biosynthesis, glycolysis and PPDK pathway increased abundance as the endosperm developed, and were predicted in an interaction network. Our data suggested the network represents an important mechanism to orchestrate carbon partitioning among starch biosynthesis, amino acid biosynthesis and glycolysis for efficient starch and protein storage. These results provide novel insights into mechanism of starch biosynthesis and its coordination with amino acid metabolism and glycolysis in cereal endosperms.

## Author contributions

TW conceived experiments, analyzed data and wrote manuscript. HY performed experiments and analyzed data.

### Conflict of interest statement

The authors declare that the research was conducted in the absence of any commercial or financial relationships that could be construed as a potential conflict of interest.
