# Supplementary material for: Proteomic Dissection of Endosperm Starch Granule Associated Proteins Reveals a Network Coordinating Starch Biosynthesis and Amino Acid Metabolism and Glycolysis in Rice Endosperms
Source: Front Plant Sci. 2016 May 25;7:707. doi: 10.3389/fpls.2016.00707 (PMC4879773; doi:10.3389/fpls.2016.00707)

Figure S1. Experimental design for CyDye label and DIGE.

A. Protein samples assign for CyDye label and DIGE. a to d represent 4 replicates for each developmental stage. Cy3 and Cy5 were used to label protein sample, and Cy2 was used to label the internal standard which was produced by mixing equal amount of each sample.

Gel	*Cy3*	*Cy5*	*Cy2*
1	10a	15a	Standard
2	15b	20a	Standard
3	20b	10b	Standard
4	10c	15c	Standard
5	15d	20c	Standard
6	20d	10d	Standard

B. 2D-DIGE images of gels designed in A. These were merged images of Cy3 (green), Cy5 (red), and Cy2 (blue) lanes.

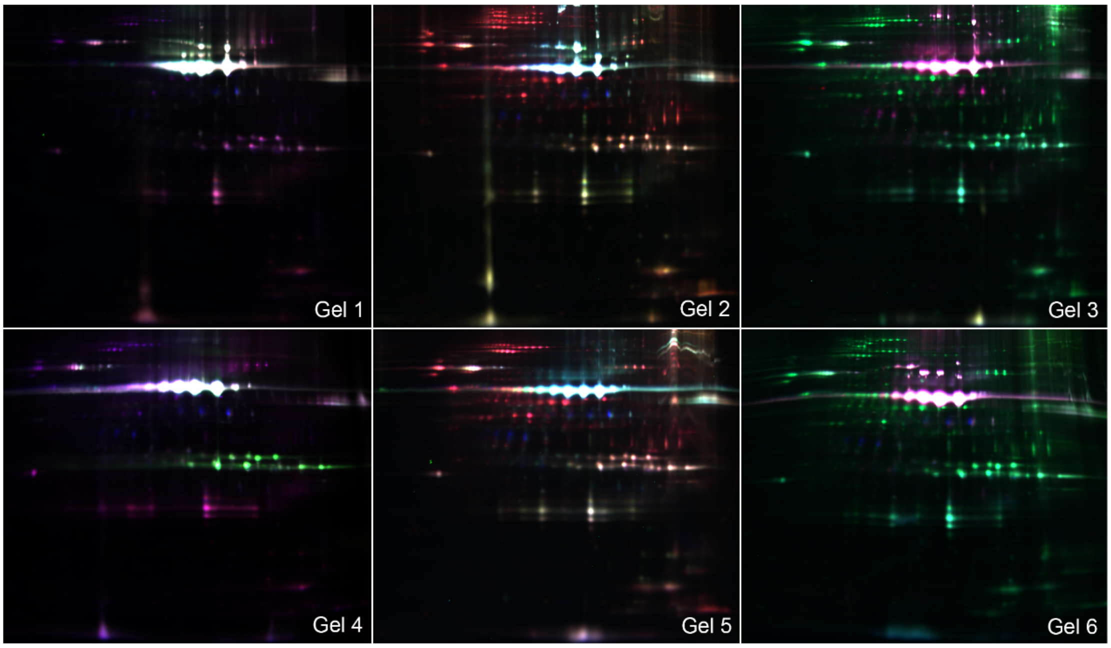

Supplement: Supplementary file 8 [file Presentation1.PDF]
