# Supplementary material for: Proteomic Dissection of Endosperm Starch Granule Associated Proteins Reveals a Network Coordinating Starch Biosynthesis and Amino Acid Metabolism and Glycolysis in Rice Endosperms
Source: Front Plant Sci. 2016 May 25;7:707. doi: 10.3389/fpls.2016.00707 (PMC4879773; doi:10.3389/fpls.2016.00707)
Supplement: Supplementary file 9 [file Presentation2.PDF]

Figure S2. MS/MS Spectra for a single confident peptide based protein identification with MALDI-TOF/TOF mass spectrometry. Proteins with only one confident matched peptide were listed in Supplementary Table S-3.

Spot 110.2

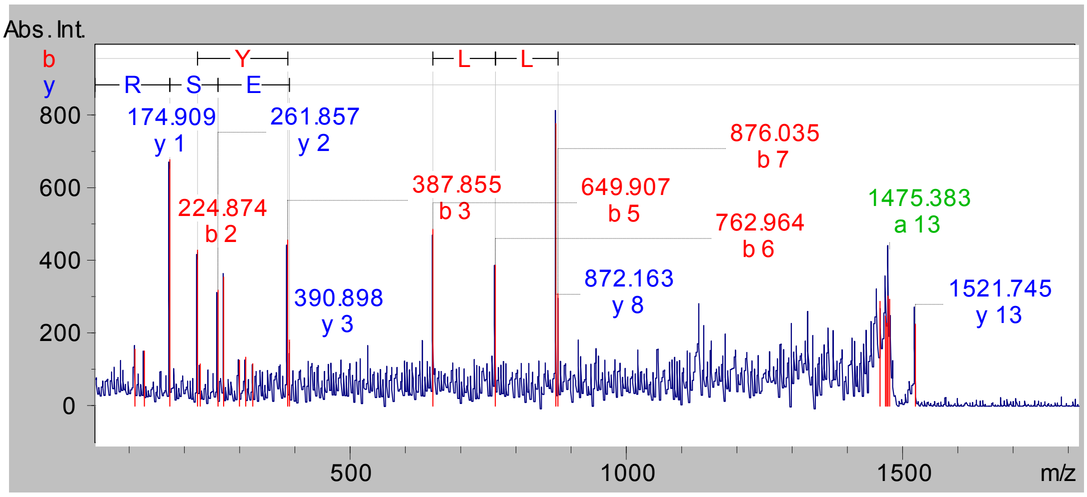

Precursor mass (m/z): 1521.7834

Sequence: R.SHYFDLLLAAESR.A

Ions score/Expect: 42/ 0.011

Spot 248.1

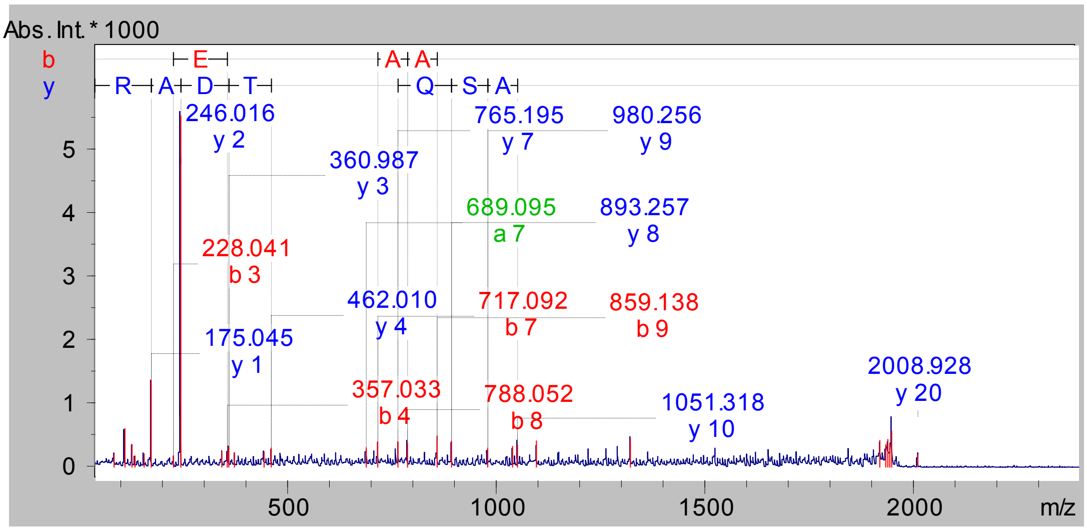

Precursor mass (m/z): 2008.9611

Sequence: M.AVGEECAAAVASQGFVTDAR.A

Ions score/Expect: 67/ 2.80E-05

Spot 248.2

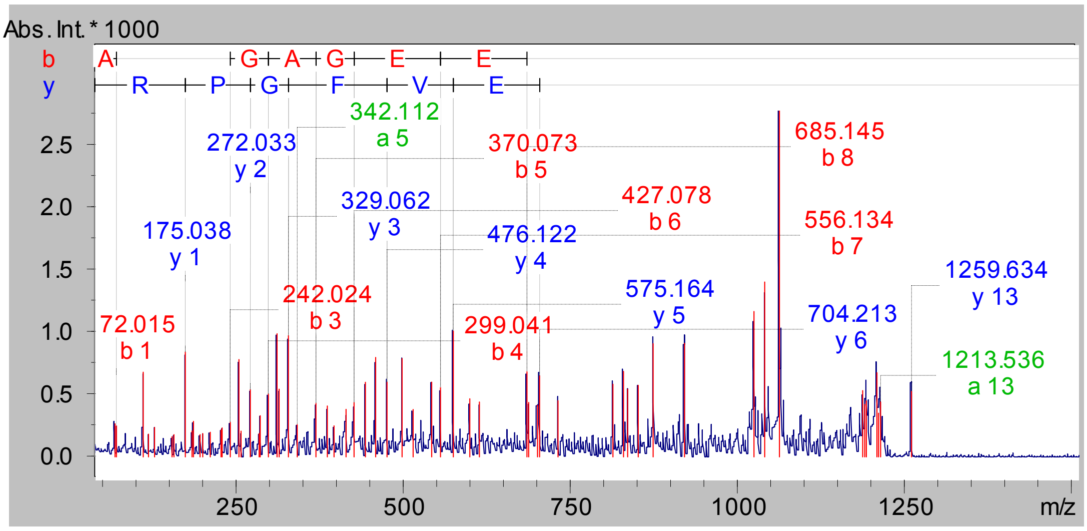

Precursor mass (m/z): 1259.6548

Sequence: R.AAVGAGEEVFGPR.R

Ions score/Expect: 53/ 1.10E-03

Spot 252

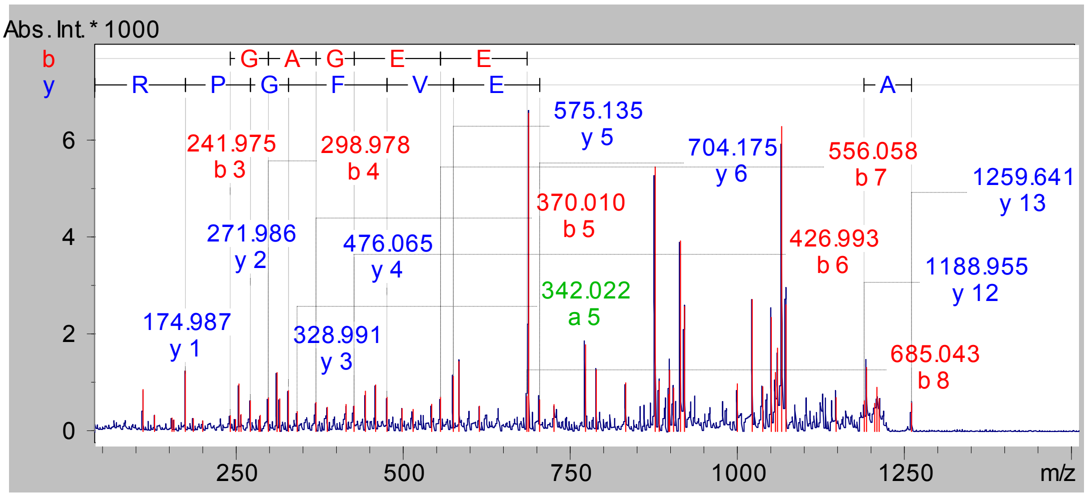

Precursor mass (m/z): 1259.6548

Sequence: R.AAVGAGEEVFGPR.R

Ions score/Expect: 53/ 1.10E-03

Spot 327

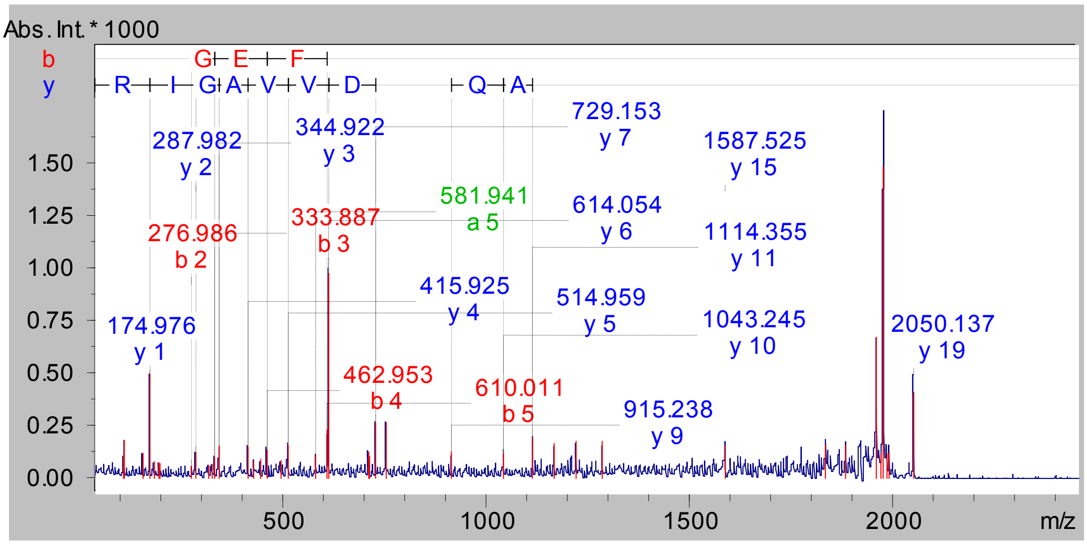

Precursor mass (m/z): 2050.158

Sequence: K.LYGEFLVNAQGEDVVAGIR.T

Ions score/Expect: 84/ 4.50E-07

Spot 506.2

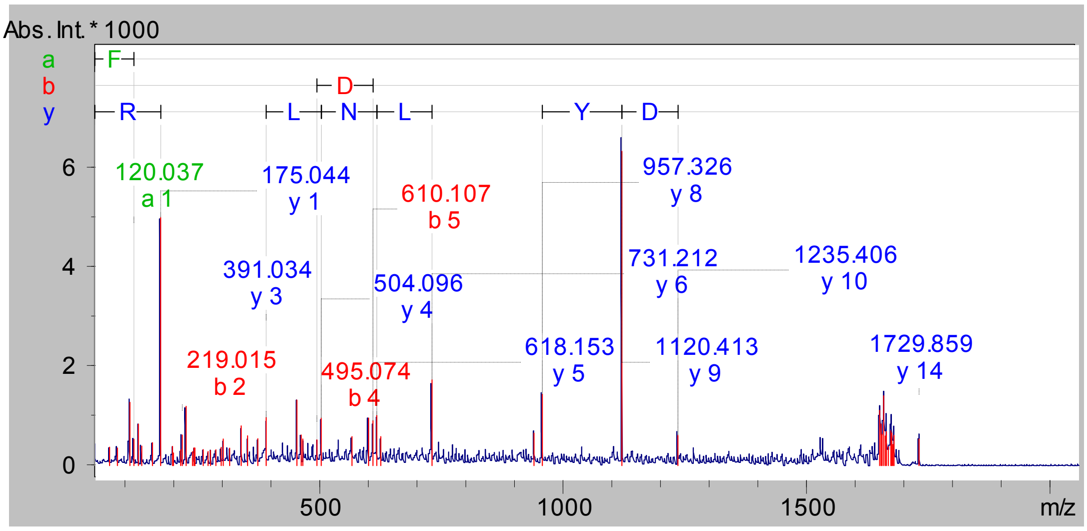

Precursor mass (m/z): 1729.8699

Sequence: R.FAFEDYPELNLSER.F

Ions score/Expect: 59/ 0.00022

527

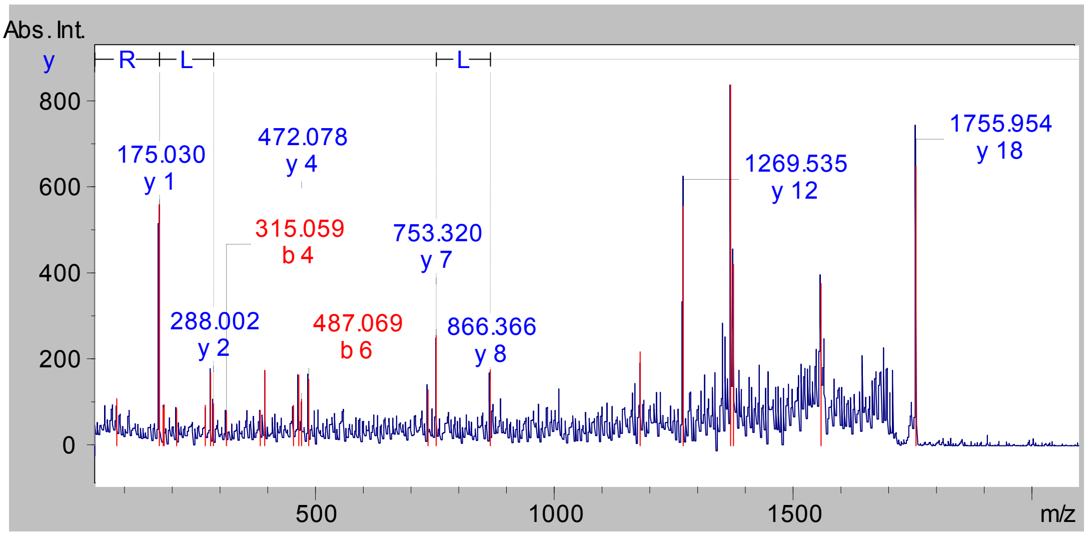

Precursor mass (m/z): 1755.9783

Sequence: K.SGGLGDVCGSLPIALALR.G

Ions score/Expect: 40/ 0.015

Spot 625.2

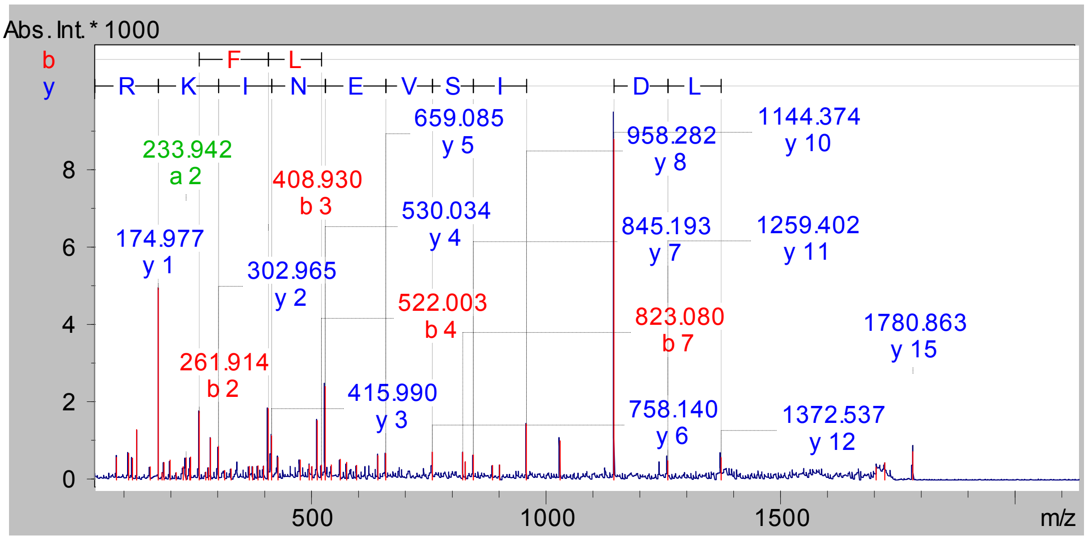

Precursor mass (m/z): 1780.8927

Sequence: R.NFFLDGEISVENIKR.F

Ions score/Expect: 101/ 1.20E-08

Spot 755.1

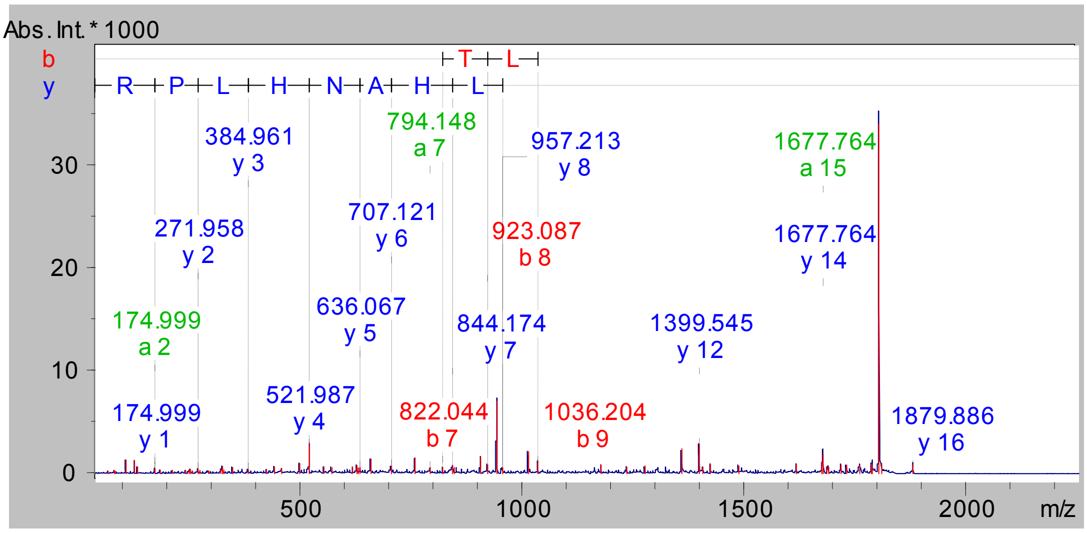

Precursor mass (m/z): 1879.9002

Sequence: R.SDYDFGHTLHANHLPR.G

Ions score/Expect: 48/ 0.0025

Spot 755.2

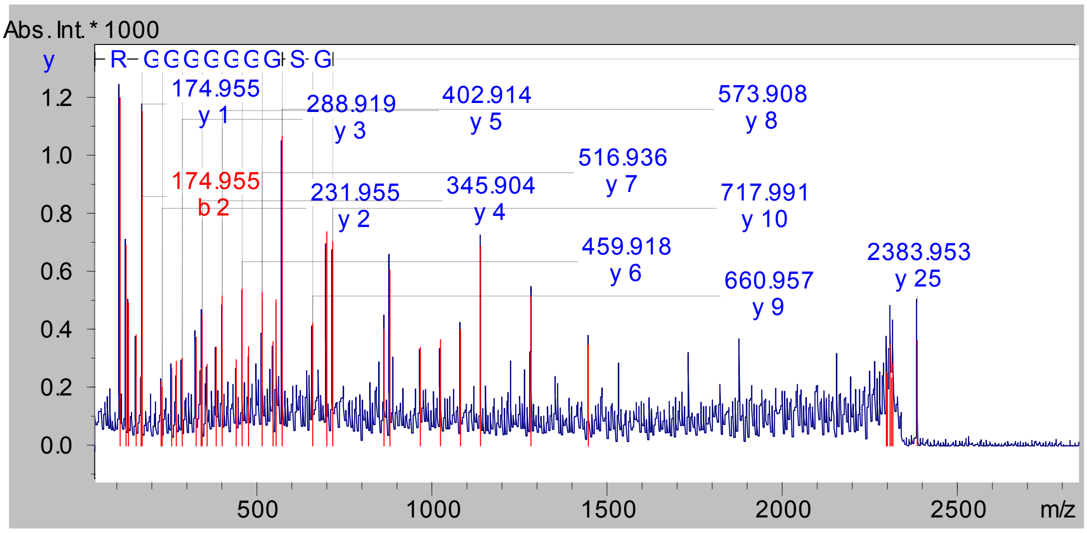

Precursor mass (m/z): 2383.9634

Sequence: M.SSWNSPYYDTSSYGAGSGGGGGGR.R

Ions score/Expect: 55/ 0.00026

Spot 758.2

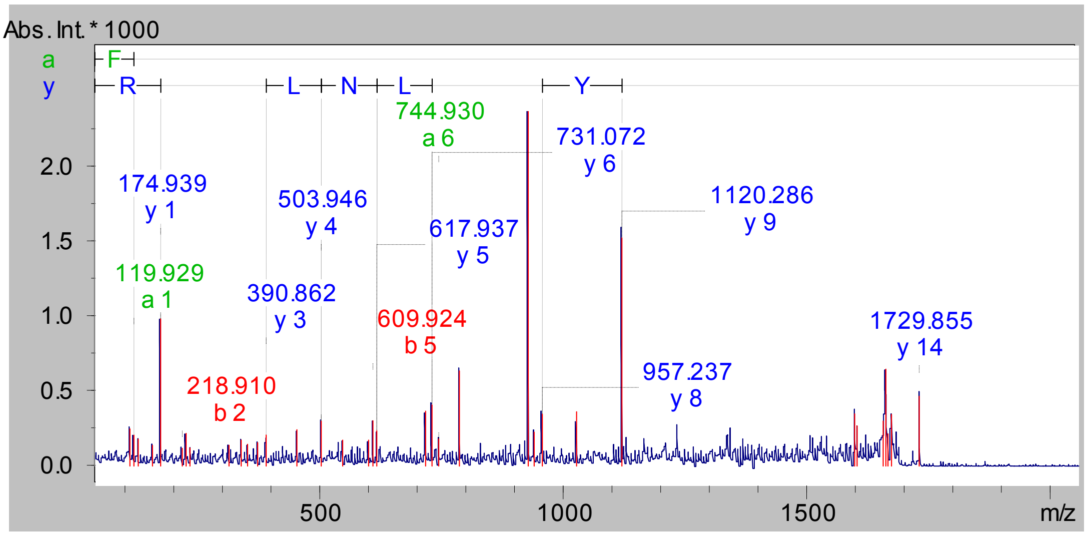

Precursor mass (m/z): 1729.8439

Sequence: R.FAFEDYPELNLSER.F

Ions score/Expect: 39/ 0.024

Spot 776.2

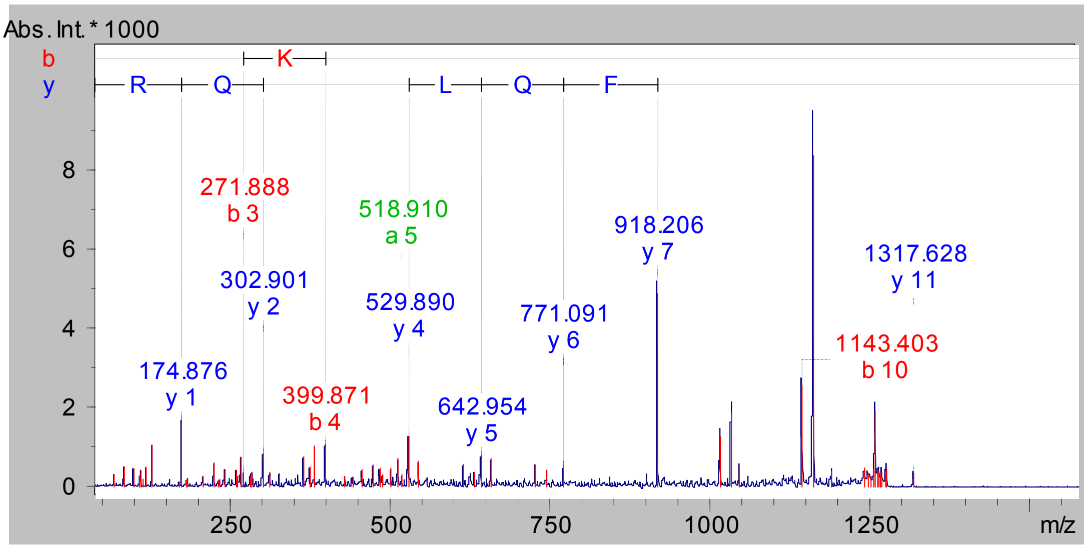

Precursor mass (m/z): 1317.6461

Sequence: K.GTLKFQLINQR.Q

Ions score/Expect: 39/ 0.032

Spot 840.2

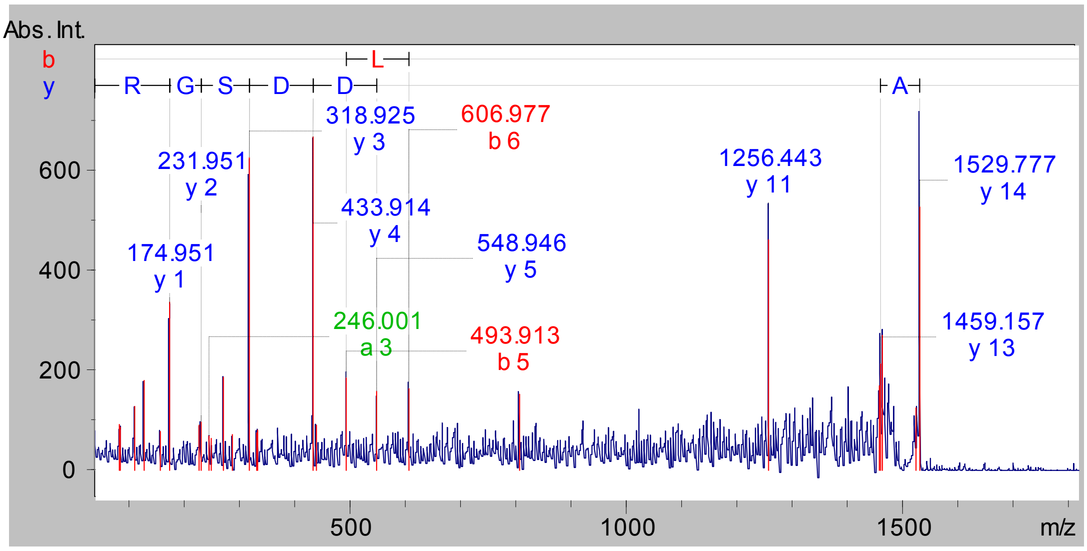

Precursor mass (m/z): 1529.7807

Sequence: R.ASDYGLVKFDDSGR.V

Ions score/Expect: 56/ 0.00052

Spot 895

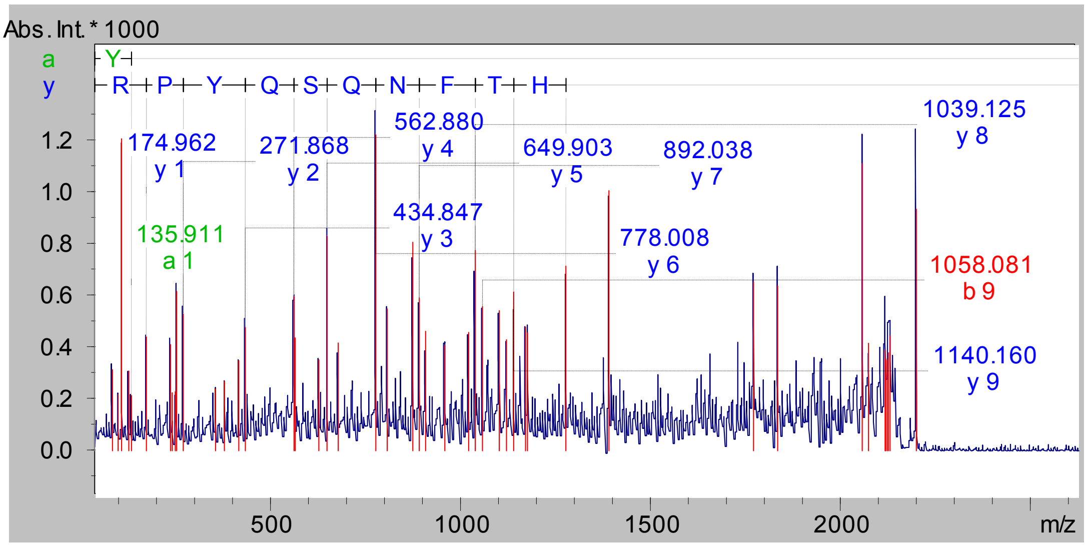

Precursor mass (m/z): 2198.1077

Sequence: K.YSNSNIEIHTFNQSQYPR.I

Ions score/Expect: 99/ 1.90E-08

Spot 934

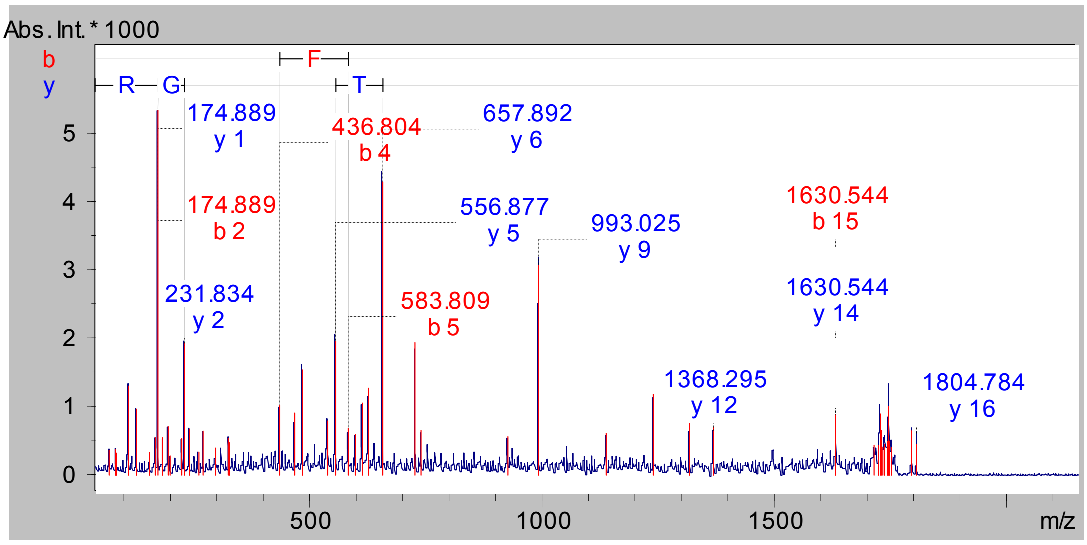

Precursor mass (m/z): 1804.7933

Sequence: R.SSFDFIDGYDTPVEGR.K

Ions score/Expect: 38/ 0.026

Spot 1123

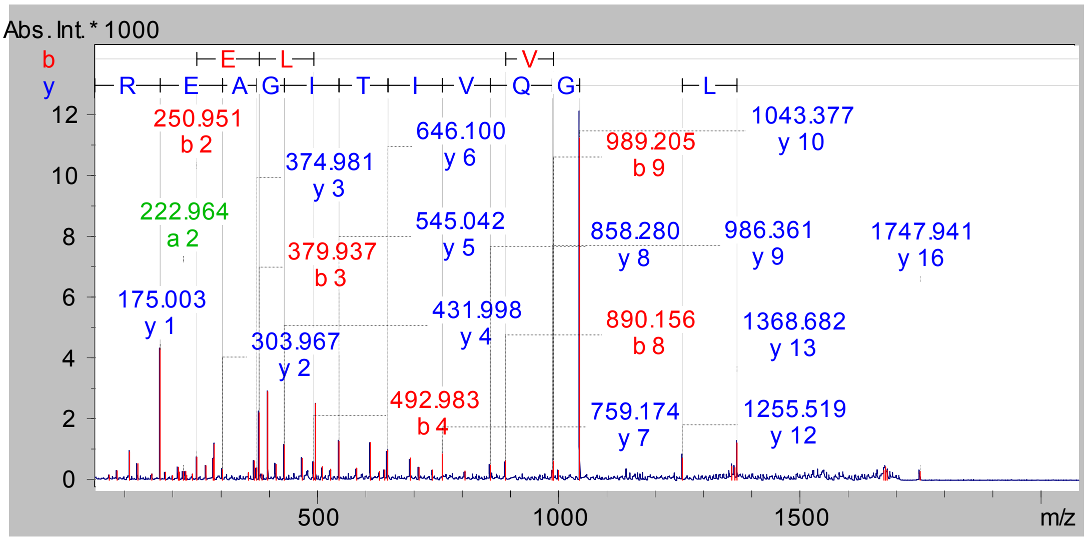

Precursor mass (m/z): 1747.9558

Sequence: K.SYELPDGQVITIGAER.F

Ions score/Expect: 101/ 1.20E-08

Spot 1428.2

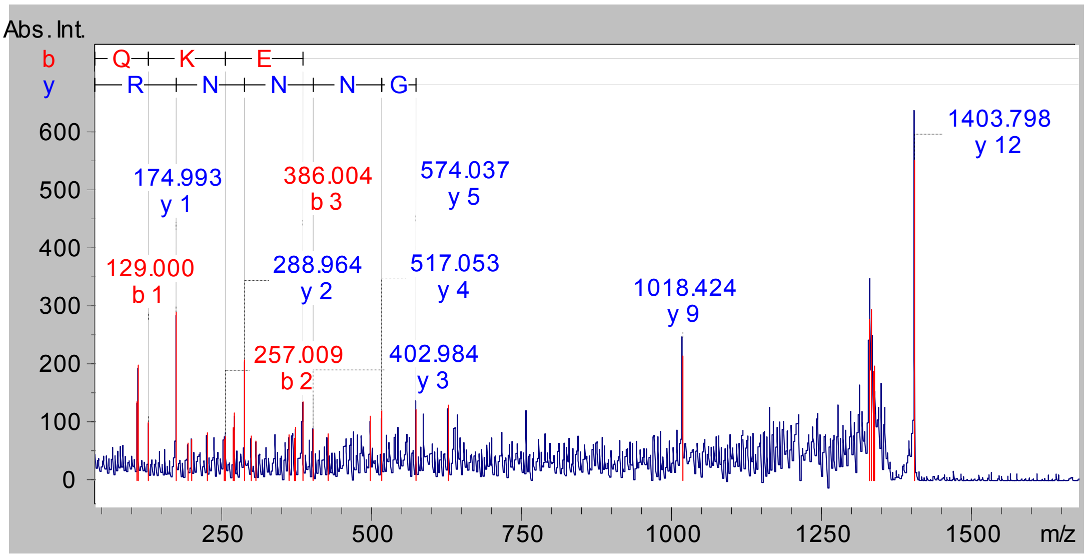

Precursor mass (m/z): 1403.8064

Sequence: R.QKEFLLAGNNNR.A

Ions score/Expect: 39/ 0.024

Spot 1543.3

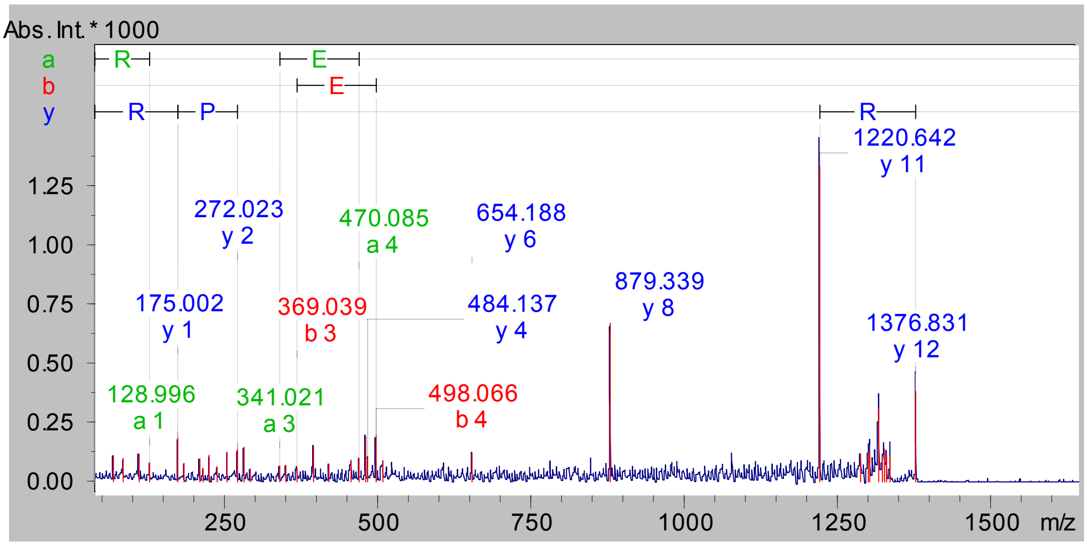

Precursor mass (m/z): 1376.8536

Sequence: R.RVIEPQGLLVPR.Y

Ions score/Expect: 41/ 0.008

Spot 1583.2

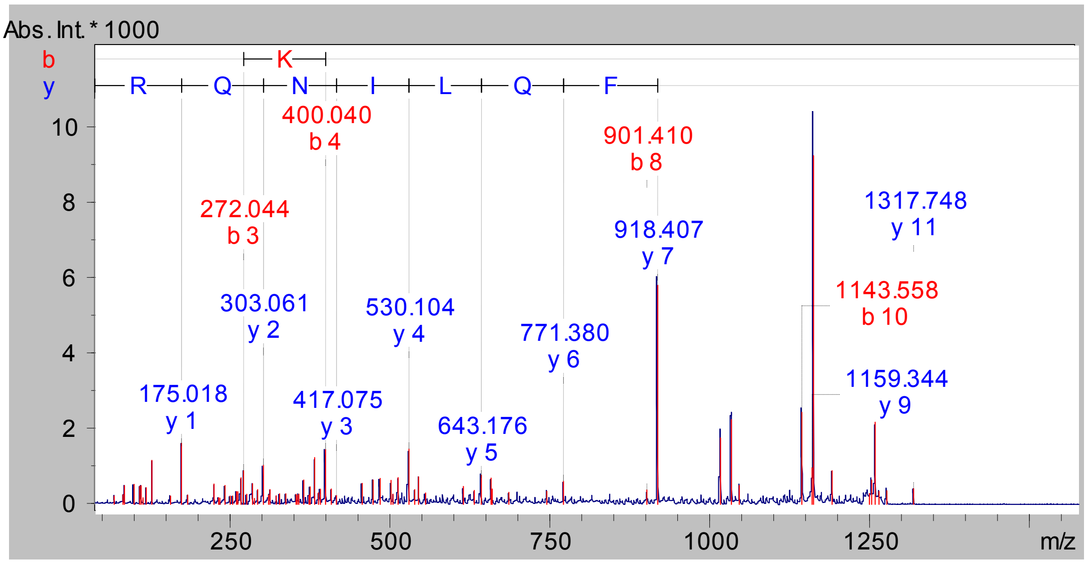

Precursor mass (m/z): 1317.767

Sequence: K.GTLKFQLINQR.Q

Ions score/Expect: 39/ 0.025

Spot 1793.1

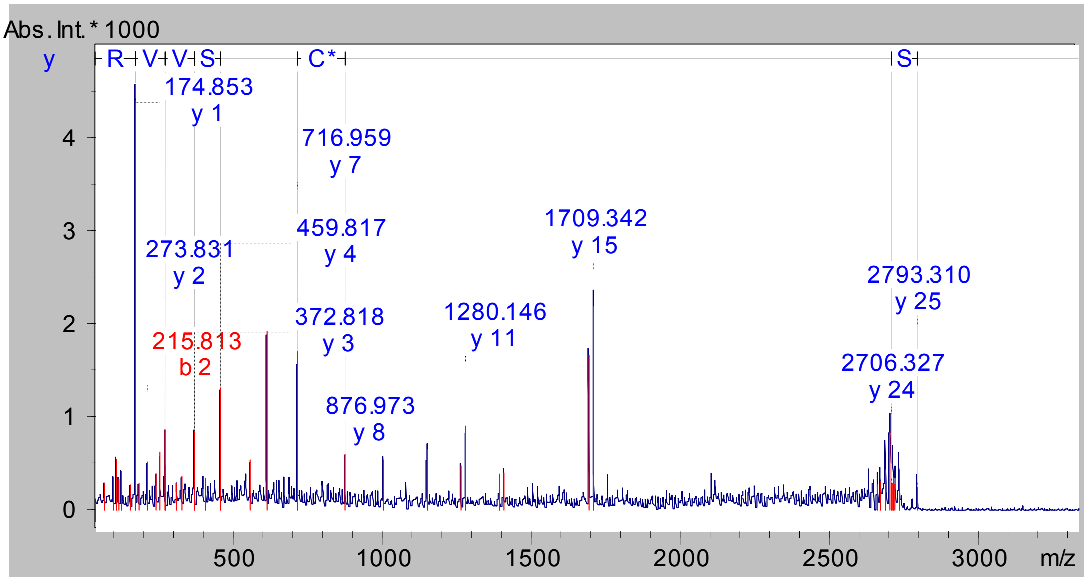

Precursor mass (m/z): 2793.3018

Sequence: R.SQAGTTEFFDVSNEQFQCTGVSVVR.R

Ions score/Expect: 42/ 0.0057

Spot 1793.2

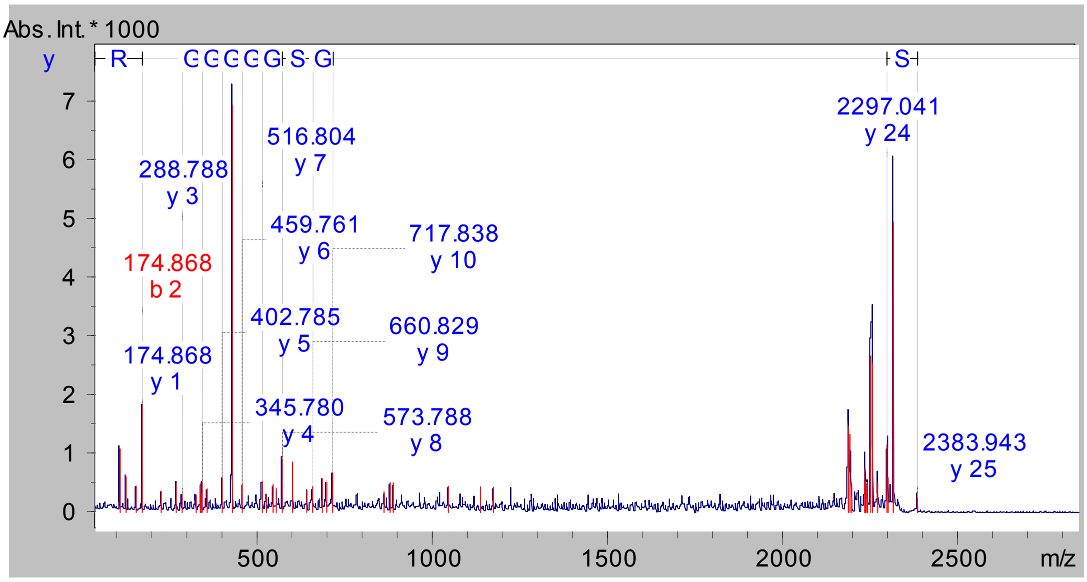

Precursor mass (m/z): 2383.9712

Sequence: M.SSWNSPYYDTSSYGAGSGGGGGGGR.R

Ions score/Expect: 47/ 0.0016

Spot 2045

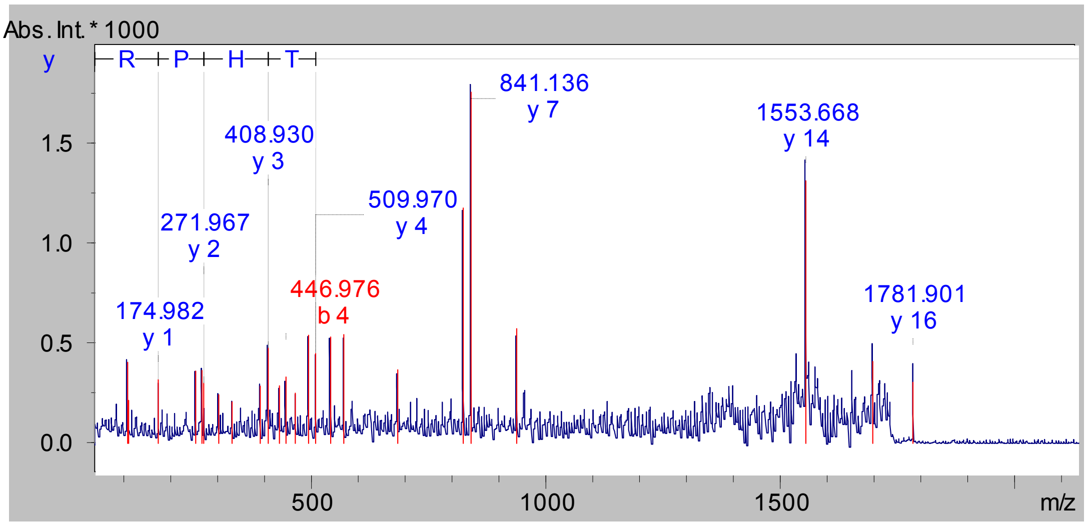

Precursor mass (m/z): 1781.928

Sequence: R.LDFAPLGLNPPHTHPR.A

Ions score/Expect: 47/ 0.0032

Spot 2096

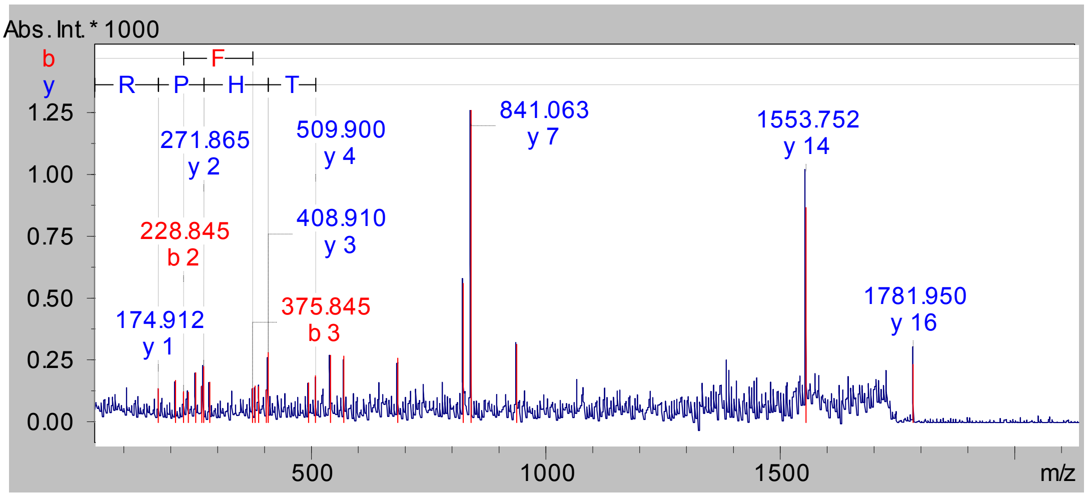

Precursor mass (m/z): 1781.9197

Sequence: R.LDFAPLGLNPPHTHPR.A

Ions score/Expect: 95/ 6.00E-08
